# Association of alcohol responsiveness and non-motor symptoms in isolated adult-onset dystonia

**DOI:** 10.1007/s00415-025-13383-8

**Published:** 2025-09-29

**Authors:** Johanna Junker, Brian D. Berman, Inke R. König, Marie Vidailhet, Emmanuel Roze, Joel S. Perlmutter, H. A. Jinnah, Norbert Brüggemann

**Affiliations:** 1 Institute of Neurogenetics, University of Luebeck, Ratzeburger Allee 160, 23538 Luebeck, SH Germany; 2 Department of Neurology, University of Luebeck, Ratzeburger Allee 160, 23538 Luebeck, SH Germany; 3 Department of Neurology, Virginia Commonwealth University, Richmond, VA USA; 4 Institute of Medical Biometry and Statistics, University of Luebeck, Luebeck, Germany; 5 Département de Neurologie, Hôpital Pitié-Salpêtrière, Assistance Publique—Hopitaux de Paris, Paris, France; 6 Sorbonne Université, Paris Brain Institute, Inserm, CNRS, Paris, France; 7 Department of Neurology, Washington University in St. Louis, St. Louis, MO USA; 8 Department of Neurology and Human Genetics, Emory University, Atlanta, GA USA

**Keywords:** Dystonia, Alcohol responsiveness, Non-motor symptoms, Pain

## Abstract

**Objective:**

About 30% of patients with isolated adult-onset dystonia report an improvement of their motor symptoms after the consumption of alcohol. In this cross-sectional study, we sought to investigate whether the observed improvement is attributable to the anxiolytic, euphoric, and analgesic properties of alcohol, rather than or in addition to its effect on dystonic movements, as psychiatric symptoms and pain frequently occur in dystonia patients and as emotional stress is a well-established trigger for symptom exacerbation.

**Methods:**

We analyzed data from 339 prospectively enrolled participants with recently diagnosed isolated dystonia (mean age: 55.2 ± 12.5 years, 228 female) of the Natural History Project of the Dystonia Coalition, a large international multicenter study. Alcohol responsiveness was determined by patients´ self-report. Symptoms of depression, as well as generalized and social anxiety, were assessed using the Hospital Anxiety and Depression Scale and the Liebowitz Social Anxiety Scale. Severity of pain was measured using question 21 of the RAND 36-Item Health Survey.

**Results:**

Participants with more severe pain reported greater response to alcohol than those with less severe pain (*p* = .004), whereas symptoms of depression (*p* = .986), generalized anxiety (*p* = .395) and social anxiety (*p* = .953) were not associated.

**Conclusion:**

Alcohol responsiveness in isolated dystonia is associated with higher levels of pain, whereas self-reported alcohol-related improvements in dystonic movements or tremor do not depend on the euphoric or anxiolytic effects of alcohol. This finding underscores the potential role of pain management in alleviating motor symptoms in dystonia.

**Supplementary Information:**

The online version contains supplementary material available at 10.1007/s00415-025-13383-8.

## Introduction

Almost one-third of patients with isolated adult-onset dystonia report an improvement of their motor symptoms after consumption of alcohol [1]. One of the discussed mechanisms is the enhancement of cerebral GABA-ergic neurotransmission, which leads to a reduction of dystonia-related disinhibition of the sensorimotor network and reduced motor symptoms [1–4]. Determinants of alcohol responsiveness in dystonia include a positive family history for movement disorders, more widespread dystonia and an earlier age of onset—possibly indicating an association of alcohol responsiveness with an underlying genetic contribution[1] as genetic dystonias are often characterized by an earlier onset and a tendency to progress to multifocal or generalized dystonia [5–9]. Furthermore, the presence of tremor, laryngeal dystonia, and/or cervical dystonia relates to a higher incidence of alcohol responsiveness [1, 10]. Interestingly, patients with cervical and laryngeal onset of dystonia commonly have generalized anxiety disorder (GAD) and those with laryngeal onset frequently also experience social anxiety disorder (SAD) [11, 12]. Additionally, symptoms of depression are a prevalent complaint in isolated adult-onset dystonia [11–13] and hereditary dystonia types as e.g. TOR1A carriers have a higher risk of recurrent major depressive disorder than non-carriers [14]. Studies on alcohol responsiveness of motor symptoms in dystonia are mostly based on self-reported or caregiver-reported information and have not been evaluated using standardized clinical assessments by movement disorder specialists [1, 10]. This raises the question of whether alcohol responsiveness relates to the anxiolytic effects of alcohol or its influence on emotional stress [15] rather than or in addition to improvement of dystonic movements [1], or whether it relates to the analgesic properties of alcohol, as dystonia is frequently associated with pain [12, 16, 17]. The reduction of anxiety and depressive symptoms through alcohol could, for example, lessen concerns about dystonia or improve motor symptoms, as emotional stress is a well-established trigger for its exacerbation [1, 7, 15]. In contrast, the analgesic effect of alcohol could, for example, indirectly improve the motor symptoms without primarily altering the dystonia-related disinhibition of the sensorimotor network. We used data from the international multicenter prospective Dystonia Coalition study to address this question.

Addressing this question is particularly important for guiding therapeutic strategies in alcohol-responsive dystonia. Should future research focus on exploring other GABA-enhancing substances, such as sodium oxybate, which mimics some effects of alcohol [18], or rather on targeted treatment of psychiatric symptoms and pain?

## Methods

### Participants

Our analysis included participants’ baseline data from the Natural History Project of the Dystonia Coalition clinical database collected across 36 international recruiting sites (Online Resource 1). Only isolated dystonia patients, aged 18 years and older with dystonia onset no more than five years prior to study enrollment were included (https://www.rarediseasesnetwork.org/cms/dystonia).

Participants of the Natural History Project answered a standardized questionnaire (Natural History Intake Form)*,* a set of validated rating scales (such as e.g. RAND 36-Item Health Survey, HADS, LSAS) and were clinically examined using a standardized video protocol. Videos were evaluated by a neurologist specialized in movement disorders and rated using the Burke-Fahn-Marsden Dystonia Rating Scale (BFMDRS). Enrollment of dystonia participants with botulinum toxin (BoNT) treatment was performed when symptoms returned, usually after three months, but never less than two months after treatment. Exclusion criteria included combined and secondary dystonia, presence of a confirmed mutation in a dystonia-related gene at the time of the analysis of our study and disorders prohibiting the completion of the questionnaire or the physical examination.

### Questionnaire and rating scales

#### Dystonia Coalition Questionnaire—Natural History Intake Form

Demographic and clinical data included sex, age, affected body regions, presence of tremor, age at onset, disease duration, mutation in dystonia-related genes in study participants or relatives, presence of other movement disorders and family history of movement disorders. Alcohol responsiveness was assessed using the question ‘Has alcohol relieved the dystonia?’ with the answer options ‘yes’, ‘no’ or ‘unknown’. Patients who answered ‘unknown’ with regard to alcohol responsiveness were also excluded.

#### Hospital Anxiety and Depression Scale (HADS)

The severity of anxiety and depressive symptoms was assessed with the HADS version 4 [19]. Each of the two subscales (HADS-A, HADS-D) contains seven questions rated on a Likert 4-point scale (0–3), each subscale yielding score ranging from 0 to 21.

#### Liebowitz Social Anxiety Scale (LSAS)

The severity of social anxiety symptoms was assessed with the LSAS [20], which is a 24-item scale divided into two subscales assessing fear/anxiety concerning performance and pertaining to social situations as well as avoidance behavior. The 24 items of the two subscales are rated on a Likert Scale from 0 to 3 yielding a maximum sum score of 144.

#### Pain

Severity of pain was assessed using question 21 of the RAND 36-Item Health Survey 1.0 [21]: ´How much bodily pain have you had during the past 4 weeks?´. The response is rated: 1 none, 2 very mild, 3 mild, 4 moderate, 5 severe, 6 very severe.

### Statistical analysis

The associations of the severity of depression, generalized anxiety, social anxiety and pain with alcohol responsiveness were assessed using binary logistic regression with alcohol responsiveness as the dependent variable and HADS-D, HADS-A, LSAS and pain severity as independent variables, including a pairwise evaluation of interactions between the independent variable pain by the independent variables HADS-D, HADS-A, and LSAS. Goodness of fit was assessed by Nagelkerke *R*^2^. As previous findings demonstrated no effect of age or sex on alcohol responsiveness in dystonia [1], we omitted adjustment for these variables in the current analysis to enhance statistical power by minimizing the number of variables.

## Results

Out of 615 participants, 12 were excluded based on clinical criteria. Of the remaining 603, 253 participants answered the question ‘Has alcohol relieved the dystonia?’ with ‘unknown’ either due to lack of alcohol consumption or a failure to remember. Out of the remaining 350 participants, 339 participants had complete data (mean age: 55.2 ± 12.5 years, female *n* = 228/67.3%) and were included in the binary logistic regression analysis (Table 1).

**Table 1:** Clinical data of the study cohort

	Alcohol responsive *n* = 83	Non-alcohol responsive *n* = 256
Sex		
Female	61.4% (51/83)	69.1% (177/256)
Male	38.6% (32/83)	30.9% (79/256)
Age	52.0 ± 12.5	56.3 ± 12.3
Main groups of dystonia		
Focal	61.4% (51/83)	62.9% (161/256)
Segmental	25.3% (21/83)	30.9% (79/256)
Multifocal/generalized	13.3% (11/83)	6.3% (16/256)
Subgroups of focal dystonia		
Cranial	3.6% (3/83)	14.8% (38/256)
Laryngeal	6.0% (5/83)	7.0% (18/256)
Cervical	48.2% (40/83)	33.2% (85/256)
Limb	3.6% (3/83)	7.8% (20/256)
Depression (0–21)	20.5% (17/83)	24.6% (63/256)
	4.6 ± 3.8	4.8 ± 4.4
Generalized Anxiety (0–21)	44.6% (37/83)	36.7% (94/256)
	6.6 ± 4.1	6.5 ± 4.4
Social Anxiety (0–144)	41.0% (34/83)	44.9% (115/256)
	28.7 ± 22.8	33.8 ± 27.3
Pain (1–6)	88% (73/83)	82.8% (212/256)
	3.3 ± 1.4	2.9 ± 1.4

Sex and type of dystonia with percentage and numbers, as well as mean age with SD of patients with and without alcohol responsiveness. Percentage and numbers of patients with symptoms of depression (HADS-D > 7), generalized anxiety (HADS-A > 7), social anxiety (LSAS > 30), and pain (RAND 36-Item Health Survey pain question > 1) shown with mean and SD for HADS-D, HADS-A, LSAS, and pain scores for patients with and without alcohol responsiveness

**Fig. 1:**
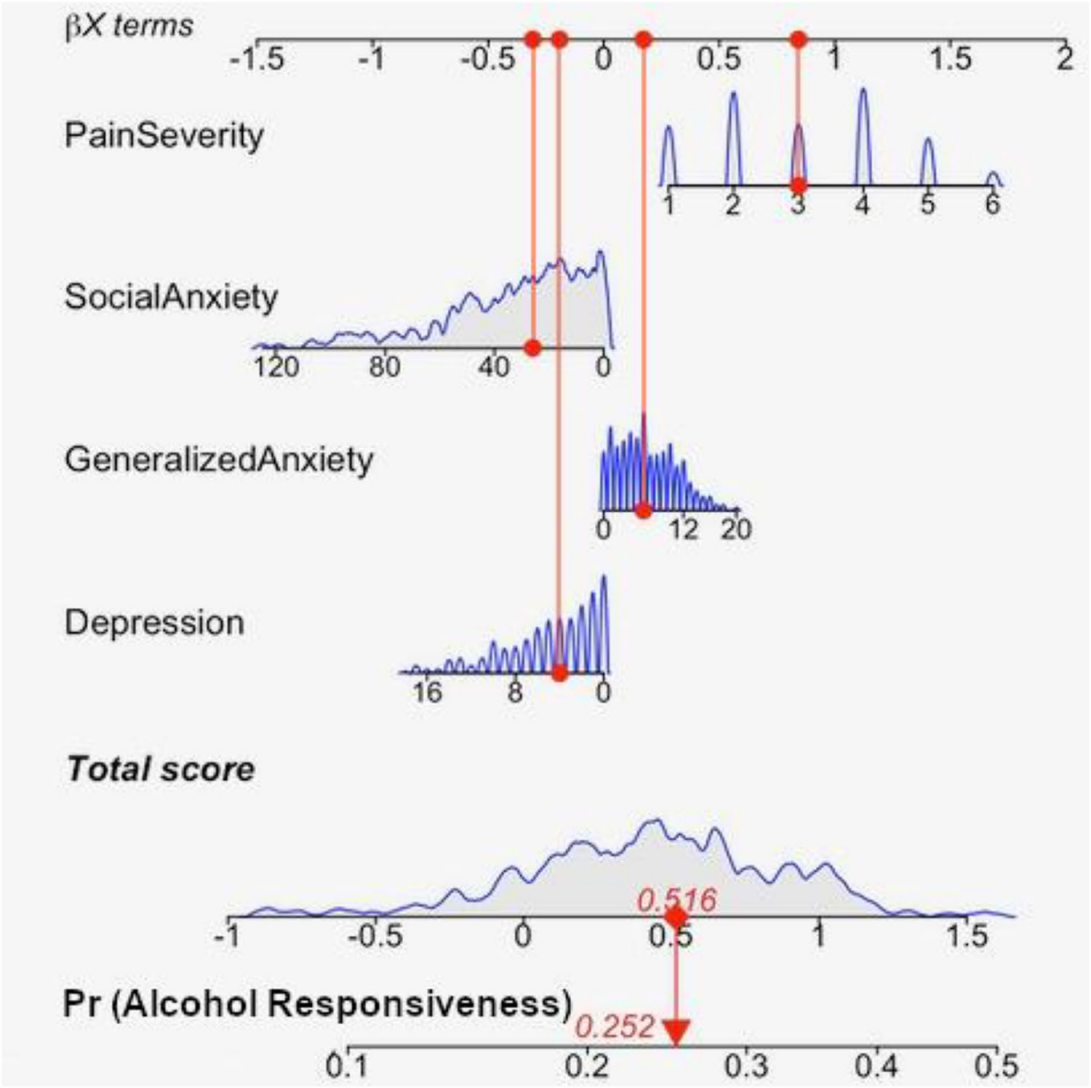
Estimation of responsiveness of non-motor signs on alcohol

A binary logistic regression analysis with severity of depressive, generalized anxiety and social anxiety symptoms as well as pain severity as independent variables showed that those with more severe pain were more likely responsive to alcohol than those with less severe pain (Nagelkerke *R*^2^ = .06; *β* = .56, SEM = .19, *p* = .004), whereas symptoms of depression (*β* = .00, SEM = .11, *p* = .986), generalized anxiety (*β* = .09, SEM = .10, *p* = .395) and social anxiety (*β* = .00, SEM = .02, *p* = .953) were not associated. There were no interactions between severity of pain and depression (*β* = −.01, SEM = .03, *p* = .681), between severity of pain and generalized anxiety (*β* = −.02, SEM = .03, *p* = .512) or between severity of pain and social anxiety (*β* = −.00, SEM = .00, *p* = .366). Figure 1 shows a nomogram representing the likelihood of alcohol responsiveness based on the severity of pain, depression, anxiety, and social anxiety.

Nomogram: Represented are the medians of all patients (red points) concerning pain severity, social anxiety, generalized anxiety and depression scores. Corresponding values of these red points as *β*(*X *− *m*) terms are summed up and delegated as total score. The level of the total score represents the likelihood of alcohol responsiveness (Pr(AlcoholResponsiveness)). The evaluation of interactions was omitted due to missing interactions of the independent variables in the initial logistic regression.

For illustration, if a patient has a median value of 3 in the pain scale (26 social anxiety, 6 generalized anxiety, 4 depression scale), the corresponding estimates are 0.843 for pain severity (−0.312 for social anxiety, 0.174 for generalized anxiety, −0.192 for depression; see upper line); these are summed to be a total score of 0.516 (second to last line), which translates into a probability of alcohol responsiveness of 0.252.

## Discussion

In this analysis of the large, multicenter Dystonia Coalition database, isolated adult-onset dystonia participants with more severe pain are more often responsive to alcohol than those with less severe pain. Alcohol responsiveness in dystonia was not associated with the severity of psychiatric symptoms (depression, generalized anxiety and social anxiety), and there was no interaction between pain and psychiatric symptoms.

The lack of an association between psychiatric symptoms and alcohol responsiveness in dystonia suggests that self-reported alcohol-related improvements of dystonia or tremor do not depend on the euphoric and anxiolytic effects of alcohol [22, 23].

Pain is a highly disabling complaint in isolated adult-onset dystonia [11, 24, 25] with a prevalence of up to 75% in those with cervical dystonia [16]. Furthermore, pain-pressure thresholds are remarkably lower in individuals with cervical dystonia compared to healthy controls [16]. Moreover, a study in healthy participants showed that a mean blood alcohol content of only 0.08% resulted in a moderate to large reduction in pain intensity ratings and that an increased blood alcohol content related to increasing analgesia [17]. One possible explanation for our findings is that the consumption of alcohol by dystonia patients reduces pain thereby lessening motor symptoms. Our results may further help explain alcohol misuse in patients with persistent pain as observed in a subgroup of dystonia patients [26].

This study has limitations: Most importantly, alcohol responsiveness in this study was evaluated through self-assessment with limited response options (yes, no, unknown). A more comprehensive investigation of this phenomenon will require both subjective and, in particular, objective assessments of the impact of alcohol consumption on dystonia-related motor symptoms (e.g., posturing, tremor, duration of improvement). Additional important parameters include the degree of alcohol responsiveness, changes in drinking habits, as well as the degree and localization of pain and its modulation by alcohol. The inclusion of a placebo-controlled group would be advisable to validate these findings.

Another limitation of our analysis is that disease duration of studied population was less than five years, which prevented the assessment of later disease stages.

Patients with a known monogenic background of dystonia were excluded to control for a potential confounder, as they may exhibit a distinct neuropsychiatric profile and response to alcohol. It should be noted as a limitation that only carriers of known mutations in dystonia-related genes were excluded, while no systematic genetic screening was performed at the time of the analysis of our study. In the meantime, genetic data from the Dystonia Coalition cohort have also been published, which—similar to previous studies in other cohorts—demonstrate that the mutation frequency in isolated dystonia cases is generally very low, whereas the likelihood of detecting causative mutations is considerably higher in combined or complex dystonias, as demonstrated in a large dystonia cohort subjected to exome sequencing [9, 27, 28]. Further, mutations in dystonia-related genes occur more frequently in generalized or multifocal dystonia, whereas they are only rarely observed in patients with focal dystonia [9, 29].

In conclusion, we found that the severity of pain related to alcohol responsiveness in dystonia, highlighting the analgesic properties of alcohol in this alcohol responsive isolated dystonia population and the potential role of pain management in alleviating motor symptoms, in addition to new therapeutic approaches with other GABAergic substances such as sodium oxybate, which may mimic some effects of alcohol [18]. However, the individual perception of improvement of dystonic movements and tremor from alcohol in adult-onset isolated dystonia patients does not relate to the severity of anxiety or depressive symptoms and there is no interaction between pain and psychiatric symptoms. These findings suggest that NMS, such as depression and anxiety, may warrant consideration and management as distinct clinical entities in dystonia responsive to alcohol.

## Supplementary Information

Below is the link to the electronic supplementary material.

Supplementary file1 Online Resource 1. Coinvestigators of the Dystonia Coalition Study Group, who had a major role in the acquisition of data (DOCX 30 KB)

## Data Availability

Anonymized data (study protocol, statistical analysis) will be shared upon request from any qualified investigator. Data will be available for 10 years.

## Declarations

### Patient consents to participate/publish

All participants gave written informed consent for study participation and for the publication of the data prior to their enrollment.
