# Supplementary material for: Association of alcohol responsiveness and non-motor symptoms in isolated adult-onset dystonia
Source: J Neurol. 2025 Sep 29;272(10):659. doi: 10.1007/s00415-025-13383-8 (PMC12477084; doi:10.1007/s00415-025-13383-8)
Supplement: Supplementary file 1 — Supplementary file1 Online Resource 1. Coinvestigators of the Dystonia Coalition Study Group, who had a major role in the acquisition of data (DOCX 30 KB) [file 415_2025_13383_MOESM1_ESM.docx]

**Association of alcohol responsiveness and non-motor symptoms in isolated adult-onset dystonia**

Johanna Junker, MD^1,2^, Brian D. Berman, MD, MS^3^, Inke R. König, PhD^4^, Marie Vidailhet, MD^5,6^, Emmanuel Roze, MD, PhD^5,6^, Joel S. Perlmutter, MD^7^, H. A. Jinnah, MD, PhD^8^, Norbert Brüggemann, MD^1,2^, for the Dystonia Coalition Investigators^9^

^1^ Institute of Neurogenetics, University of Luebeck, Luebeck, Germany

^2^ Department of Neurology, University of Luebeck, Luebeck, Germany

^3^ Department of Neurology, Virginia Commonwealth University, Richmond, VA, USA

^4^ Institute of Medical Biometry and Statistics, University of Luebeck, Luebeck, Germany

^5^ Département de neurologie, Hôpital Pitié-Salpêtrière, Assistance Publique - Hopitaux de Paris, Paris, France

^6^ Sorbonne Université, Paris Brain Institute, Inserm, CNRS, Paris, France

^7^ Department of Neurology, Washington University in St. Louis, St. Louis, MO, USA

^8^ Department of Neurology and Human Genetics, Emory University, Atlanta, GA, USA

^9^ see Suppl. 1/ Online Resource 1

**Corresponding Author:**

Norbert Brüggemann MD

Dept. of Neurology and Institute of Neurogenetics, University of Lübeck

Ratzeburger Allee 160

Lübeck, SH, 23538, Germany

Phone +49-451-500 43420

Fax +49-451-500 43404

[norbert.brueggemann@uni-luebeck.de](mailto:norbert.brueggemann@uni-luebeck.de)

https://orcid.org/0000-0001-5969-6899

**Online Resource 1**

Coinvestigators of the Dystonia Coalition, that contributed participants included in this manuscript:

| **Name** | **Affiliations** |
| --- | --- |
| Adler C. H., MD, PhD | Mayo Clinic (Rochester, MN, USA) |
| Agarwal P., MD, FAAN | Booth Gardner Parkinson Care Center (Kirkland, WA, USA) |
| Barbano R. L., MD, PhD | University of Rochester (Rochester, NY, USA) |
| Berardelli A., MD | Universitá Degli Studi Di Roma „La Sapienza“ (Rome, Italy) |
| Berman B., MD | University of Colorado (Aurora, CO, USA) |
| Bhatia K., MD, DM, FRCP | University College of London (London, UK) |
| Blumin J., MD | Medical College of Wisconsin (Milwaukee, WI, USA) |
| Brashear A., MD | Wake Forest Health Sciences (Winston-Salem, NC, USA) |
| Chouinard S., MD | CHUM, University of Montreal (Montreal, Quebec, Canada) |
| Comella C. L., MD | Rush University (Chicago, IL, USA) |
| Espay A., MD | University of Cincinnati (Cincinnati, OH, USA) |
| Fox S. H., MD, PhD | Toronto Western Hospital (Toronto, Ontario, Canada) |
| Fung V. S. C., MD | Westmead Hospital (Westmead, New South Wales, Australia) |
| Grill S., MD, PhD | Parkinsons and Movement Disorders Center of Maryland (Elkridge, MD, USA) |
| Hallett M., MD | National Institutes of Health (Bethesda, Maryland, USA) |
| Harlow T. L., MD | Sanford Health - Fargo (Fargo, ND, USA) |
| Jankovic J., MD | Baylor College of Medicine (Houston, TX, USA) |
| Jinnah H. A., MD, PhD | Emory University (Atlanta, GA, USA) |
| Khemani P., MD | University of Texas Southwestern (Dallas, TX, USA) |
| Klein C., MD | University of Luebeck (Luebeck, Germany) |
| LeDoux M. S., MD, PhD | University of Tennessee (Memphis, TN, USA) |
| Leegwater-Kim J., MD, PhD | Lahey Clinic (Burlington, MA, USA) |
| Malaty I., MD | University of Florida (Gainesville, FL, USA) |
| Mari Z. K., MD | Johns Hopkins University (Baltimore, MD, USA) |
| Nahab, F. B., MD | University of California San Diego (La Jolla, CA, USA) |
| Perlmutter J., MD | Washington University in St. Louis (St. Louis, MO, USA) |
| Reich S., MD | University of Maryland (Baltimore, MD, USA) |
| Richardson S. P., MD | University of New Mexico (Albuquerque, NM, USA) |
| Severt L., MD | Beth Israel Medical Center (New York, NY, USA) |
| Shih L., MD | Beth Israel Deaconess (Boston, MA, USA) |
| Stover N., MD | University of Alabama (Tuscaloosa, AL, USA) |
| Suchowersky O., MD | University of Alberta (Edmonton, Alberta, Canada) |
| Testa C. M., MD, PhD | Virginia Commonwealth University (Richmond, VA, USA) |
| Truong D., MD | Parkinson`s and Movement Disorders Institute (Fountain Valley, CA, USA) |
| Uc, E. Y., MD | University of Iowa (Iowa City, IA, USA) |
| Vidaihet M., MD;  Flamand-Roze E., MD | Hospital de la Salpetriere (Paris, France) |
| Xie T., MD, PhD | University of Chicago (Chicago, IL, USA) |
